# Patient Perceptions of a Population Health Management Program to Improve Kidney Care: Optimizing care in CKD

**DOI:** 10.1016/j.xkme.2025.101025

**Published:** 2025-05-15

**Authors:** Linda-Marie U. Lavenburg, Susan M. Devaraj, Ambreen Gul, Melanie R. Weltman, Balchandre Neilesh Kenkre, Flor de Abril Cameron, Jane O. Schell, Megan E. Hamm, Manisha Jhamb

**Affiliations:** 1 Renal-Electrolyte Division, University of Pittsburgh Medical Center, Pittsburgh, PA; 2 Department of Pharmacy & Therapeutics, University of Pittsburgh School of Pharmacy, Pittsburgh, PA; 3 Center for Biostatistics and Qualitative Methodology, University of Pittsburgh, Pittsburgh, PA; 4 Division of Internal Medicine, Section of Palliative Care and Medical Ethics, University of Pittsburgh Medical Center, Pittsburgh, PA

**Keywords:** Kidney disease, education, population health management, patient perspectives

## Abstract

**Rationale & Objective:**

A population health management intervention for a pragmatic cluster randomized control trial (Kidney CHAMP) aimed to improve care and outcomes in patients with chronic kidney disease (CKD) at high-risk of progression to dialysis dependence but not seeing a nephrologist. The Kidney CHAMP intervention provided comanagement support to primary care providers by nephrology electronic-consult, pharmacist-directed medication reconciliation, and nurse-delivered CKD patient education. We sought to learn patient perceptions of Kidney CHAMP intervention and whether the intervention improved their understanding of CKD.

**Study Design:**

An ancillary study of Kidney CHAMP using qualitative methods.

**Setting & Participants:**

Participants were sampled from Kidney CHAMP intervention group using 3 predefined strata (racial/ethnic minorities, low socioeconomic status, and multimorbidities) from May 2021 to February 2022.

**Analytical Approach:**

We conducted semistructured televideo or telephone interviews that were transcribed and then inductively coded by 2 data analysts until thematic saturation was reached. Conventional content and thematic analyses were performed.

**Results:**

In 45 patient interviews (mean age 75 ± 8 years, 44% women, 9% non-White race, and 59% low socioeconomic status), we identified 4 themes. First, patients expressed support for CKD comanagement by the primary care providers (PCPs) and nephrology team. Second, education sessions had variable effect on improving patients’understanding of CKD and its health implications. Third, patients’ self-efficacy and understanding of CKD management varied and was influenced by their understanding of its health implications. Fourth, patients appreciated education sessions and wanted more frequent sessions and actionable individualized guidance.

**Limitations:**

Low representation of non-White individuals, recall bias, and lack of validated measures for health literacy, patient knowledge, and activation.

**Conclusions:**

Patients with CKD who are managed by their PCP were supportive of remote comanagement by a nephrologist. Patients perceive some aspects of CKD health education to be beneficial; however, more effective approaches to communicating risk of CKD development and progression are needed.

## Plain-Language Summary

In this ancillary qualitative study, we aimed to learn patient perspectives on a population health management approach to kidney disease comanagement between primary care providers and a multidisciplinary nephrology team. Patient interviewees were supportive of communication between primary care providers and the nephrology specialists, and most appreciated nurse-provided education sessions. However, despite exposure to the same standardized kidney education, interviewees demonstrated variable understandings of kidney disease and its health implications. Suggestions to improve the educational sessions included providing actionable individualized guidance and more frequent sessions.

Approximately 14% of United States adults aged >30 years have chronic kidney disease (CKD).^1^ Most patients with nondialysis dependent CKD are managed by primary care providers (PCPs).^2^, ^3^, ^4^ However, PCPs may not have the specialized training, time, capacity, or system-based resources to optimally manage patients with CKD, leading to gaps in CKD care and increased risk of CKD progression and complications, such as crash start dialysis.^3^^,^^5^ Innovative solutions are needed to address potential gaps in CKD care by providing multifaceted support to PCPs and patients.^6^^,^^7^ Population health management (PHM), the process of systematically facilitating health care delivery to improve the health of a specific population of individuals, is an appealing approach to optimizing CKD care.^8^^,^^9^ PHM identifies patients who present with a specific risk profile and leverages electronic health records (EHRs) to provide clinical monitoring and intervention.^10^ Primary care organizations and nephrologists alike have recognized the potential to use EHR-based PHM to improve CKD care.^11^

To understand the effectiveness and scalability of PHM interventions, it is imperative to understand patient perspectives around their experiences with these approaches. This is particularly important given that certain aspects of care, such as medication adherence or behaviors such as dietary intake, will rely on patient self-management.^12^^,^^13^ Patient perspectives can provide insight into the acceptability of PHM intervention components and guide improvement of educational support for patients. Patient input is also needed to better understand program accessibility to persons who may experience social or health care inequities.

The Kidney Coordinated HeAlth Management Partnership (Kidney CHAMP) trial was designed to test an EHR-based PHM approach to improve care in patients with CKD managed by PCPs without a nephrology consultant and at high-risk of progressing to end-stage kidney disease.^14^ Patients were considered high-risk of progression to end-stage kidney disease if they met any of the following 3 criteria: (1) estimated glomerular filtration rate (eGFR) 15-29 mL/min/1.73m^2^; (2) 4-variable Kidney Failure Risk Equation 5-year risk of ESKD ≥ 5%; or (3) rapid eGFR decline ≥ 5mL/min/1.73m^2^ per year, based on 2 eGFR values at least 12 months apart. Two important aspects of Kidney CHAMP intervention were as follows: (1) to provide nephrology specialist and pharmacist support to PCPs for comanagement of CKD and (2) to provide patient education sessions to help increase patient awareness, engagement, and self-management for CKD. A critical element before disseminating Kidney CHAMP to other real-world practice settings is to learn the experiences of patients and PCPs impacted by the intervention. The optimizing care in CKD (OPTIMIZE CKD) ancillary study aimed to understand the barriers and facilitators of the intervention’s effectiveness in various settings and with diverse patient groups from the patient and PCP perspective and guide refinements to the program that promote accessibility to vulnerable patient groups. We are presenting the patient perspectives of Kidney CHAMP obtained through the OPTIMIZE CKD study.

## Methods

We conducted a qualitative research study using semistructured video or telephone interviews of patients enrolled in Kidney CHAMP who were randomized to PCP practices receiving the PHM intervention. The multifaceted intervention bundle included nephrology guidance, pharmacist-led medication management, and CKD education for patients. Nephrology targeted automated e-consults provided concise and actionable recommendations to PCPs for reducing cardiovascular morbidity, CKD progression, and improving medication safety. Pharmacists completed a comprehensive medication review focusing on medication safety, ease of use, and affordability. Nurse educators used telemedicine (audio or video per patient preference) to provide personalized education to patients using standardized material from National Kidney Foundation and Center for Disease Control resources.^15^^,^^16^ The nurse educators were experienced nurses, trained by study nephrologists on CKD specific topics and communication skills, which included an audit and feedback on education sessions. The educational material covered by nurse educators included topics on CKD diagnosis and stages, risk factors and importance of hypertension and diabetes control, complications including increased risk of cardiovascular disease, management topics including dietary modification and physical activity, and discussions of complications and kidney replacement therapy options including medical management without dialysis when appropriate. Patients were also advised on medication adherence and avoidance of over-the-counter nephrotoxic medications. They were also advised to look out for common side effects and how to minimize them (eg, sick day rule for diuretics), report them to PCP, and discuss optimal medications with PCP. During follow-up, e-consults, medication reviews and education sessions were conducted every 4-6 months.

### Recruitment

#### Eligibility Criteria

All patients in the intervention arm of the Kidney CHAMP study who attended at least 1 CKD education session were eligible.^14^^,^^17^ Using selective recruitment across predefined strata, we randomly selected 15 patients from racial/ethnic minorities, 15 with low socioeconomic status (using median household income based on zipcode) and 15 with multimorbidities (using median Charlson comorbidity index score) and conducted interviews to reach thematic saturation. These categories were not mutually exclusive. All patients provided informed consent, and the study was approved by the University of Pittsburgh institutional review board.

#### Qualitative Research Methodology

All aspects of this qualitative research project—data collection, transcription, and analysis—were conducted by experienced, trained interviewers and analysts at the Qualitative, Evaluation, and Stakeholder Engagement research group (QualEASE) at the University of Pittsburgh. The primary and secondary coders were trained and supervised by the qualitative methodologist expert (MH). This study followed a qualitative description approach, meaning that our goal was to collect participant opinions and experiences on a range of topics and summarize the content of those interviews, abstracting to the level of common themes but not to the level of social theory; this is a common approach in qualitative studies conducted in Medicine.^18^^,^^19^

#### Data Collection

The study team developed a semistructured interview guide focused on patient understanding of CKD, symptoms and current/future effects on their health, educational sessions with the Kidney CHAMP nurse or their physician, and opinions on a PCP-nephrologist electronic consultation. (Item S1) Although patients were called by a pharmacist, this interaction was minimal for medication reconciliation, and thus was not the focus of the interviews. The initial interview guide was pilot tested on 2 patients, and refined for clarity.

Participants were interviewed by televideo or telephone, based on interviewee preferences or capabilities from May 2021, to February 2022. After the interview, audio files were transcribed verbatim with identifying details redacted.

#### Data Analysis

An experienced QualEASE analyst inductively developed a codebook, which was reviewed by the study team to ensure that, in addition to reflecting the content of the interviews, it reflected relevant constructs of interest. Two trained analysts (BNK and FAC) from QualEASE applied the codebook to an initial set of 5 transcripts, and then finalized the codebook after discussion of definitions and criteria for the codes. Intercoder reliability was established using an iterative process by the 2 coders who independently reviewed 10 transcripts and used Brennan & Prediger Kappa scores and discussed adjudication of codes with methodologist expert until full agreement. The initial mean κ score was 0.69, which prompted a revision of codebook to consolidate a handful of very rarely used, fine-grained codes. Subsequent coding for 15 transcripts using the new codebook resulted in full agreement. The primary coder (BNK) then completed coding of all remaining transcripts. Conventional content analysis was first conducted to summarize interview content, and then thematic analysis was conducted to group concepts into themes and subthemes. NVivo software was used for data management.

## Results

Forty-five patients with CKD were interviewed. Patients had a mean ± standard deviation age of 75 ± 8 years and eGFR 37 ± 8 mL/min/1.72m^2^ (eGFR range, 19-61 mL/min/1.72m^2^). Twenty of these patients were women, 25 were men, 4 were non-White, 26 had a median household income <$45,023, and 34 had a Charlson comorbidity index value of ≥8 (Table 1). We identified 4 major themes about patients’ views regarding PCP collaboration with nephrologists and the ability of kidney education to improve patients’ understanding of the causes and risks of CKD. Representative quotes supporting each theme are presented in Table 2.

**Table 1:** Patient Participant Characteristics

Characteristics	Patients (N = 45)
Mean age (y) ± SD	75 ± 8
Female sex, n (%)	20 (44)
Non-White race, n (%)	4 (9)
eGFR (mL/min/1.73m^2^) ± SD	37 ± 8
aMedian household income < $45,023, n (%)	26 (59)
aCharlson comorbidity index ≥8, n (%)	34 (77)
Diabetes, n (%)	33 (73)
Hypertension, n (%)	45 (100)
bCardiovascular disease, n (%)	41 (91)

Abbreviations: eGFR, estimated glomerular filtration rate.

a Cut-offs are the median value for the cohort.

b Cardiovascular disease identified by electronic health record extracted diagnosis codes.

**Table 2:** Representative Quotes for Each Theme.

Subtheme	Exemplary quotes representative of themes and subthemes
**Theme 1:** patients were supportive of collaborative comanagement support from nephrologist to their PCP	
**Subtheme 1a:** patients wanted their PCPs to seek nephrologist input and maintain close communication	**Quote 1** (male, age unknown, CKD 3b) …I’m sure he (PCP) would indicate that I need to see somebody if he thought it was necessary…
	**Quote 2** (male, 68, CKD 4) …he’s a primary care doctor, which he knows a lot of about much. But, a kidney doctor, that’s what they specialize in, so they would know even more. So, yes, I would prefer that he would talk to, someone that knows more than what he does.
	**Quote 3** (female, 72, CKD Stage 3b) I mean, closer contact… with the PCP and my endocrinologist and the specialist… I’m sure they have to know everything… and they specialize in that.
	**Quote 4** (male, 68, CKD 3B) I’ve got a PCP, I got the kidney doctor, and then I’ve got a cardiologist, and they all have the ability to change my medications. When you have three people and possibly four people in there, you know, deciding to make changes…I’m not sure all of ‘em are talking with each other
	**Quote 5** (female, 82, CKD 3b) I don’t know why I have to go to the nephrologist, cuz he has all my lab work there… I thought it was a waste of time, whenever my, my PCP has the same…information… I just don’t understand why I am going to both of them…
**Theme 2:** education sessions had variable effect on improving patients’understanding of CKD and its health implications	
**Subtheme 2a:** spectrum of knowledge about CKD and its health implications	**Quote 6** (female, 64, CKD 3b) Well, I’m thinking that it’s bad, for your health and eventually, if your kidneys go, you have to go on dialysis… I don’t know what causes it (CKD)… it doesn’t really affect me in-really in any way…I feel healthy…So, it’s not really bothering me yet.
	**Quote 7** (male, age unknown, CKD 3a) I think the worst thing that could happen is you’d have to go on dialysis. If you’re lucky, you could have a kidney transplant.
	**Quote 8** (male, age unknown, CKD 3b) I do not understand what causes the disease. … And I know that it usually ends up in death…Well, [I have] a lot of [symptoms]. I have to urinate many times, more often than I used to.
	**Quote 9** (female, 80, CKD 3b) …chronic back pain. I would think that that would occur, since the kidneys are located in-kind of the small of my back.
	**Quote 10** (female, 56, CKD 3b) Well, there’s different-different causes. You could have cystic kidney disease, um, that’s hereditary. Um, and then, uh, your kidneys can just malfunction because you’re not-you’re taking medicine that affects your kidneys or you’re eating food that affects your kidneys.
	**Quote 11** (male, 67, CKD 3b) My understanding is it’s a function of the kidneys to, purify your blood and as for me, I have lost a kidney… I have to watch my diet and if something were to go wrong, I could die from it.
	**Quote 12** (female, 73, CKD 3b) I think the diabetes maybe is what caused it and the high blood pressure.
	**Quote 13** (male, 78, CKD 3b) My blood count or something was low. Something to do with my blood. I don’t know [how I was diagnosed]…[I have Stage] 5, I think.
	**Quote 14** (male, age unknown, CKD 3b) From my understanding about the chronic kidney disease is that, …it’s how your kidneys has been treated throughout the years…your way that you eat or the foods that you might consume…the chronic disease… enables you not to be able to go to the bathroom…
	**Quote 15** (female, 82, CKD 3b) You know, you know, that’s a mystery to me…maybe not enough fluids you’re not drinking. I have no idea.
**Subtheme 2b:** poor awareness of CKD and low priority	**Quote 16** (female, 80, CKD 3b) He [PCP] just kinda come up with a diagnosis one day. It was on the report… I got so many other problems, that it’s kind of taken a back seat.
	**Quote 17** (female, 71, CKD 3b) I wasn’t even aware I had kidney disease till not long ago…when I left my doctor’s office, I saw on there grade 3 or 4 chronic kidney disease, and I asked-and the next time I went, I asked him about it, and he said, oh, yeah, that’s something that happens with diabetics.
	**Quote 18** (female, 68, CKD 3) I was in the doctor’s office, uh, last week and, uh, he wasn’t concerned about it. I didn’t know anything was wrong until they told me about my blood level.
	**Quote 19** (female, 74, CKD 3b) I was never told until the last time I visited her… She said, well, you do know you have kidney disease. And I said, no, I do not know because nobody has ever told me.
**Theme 3:** patients’ self-efficacy and understanding of CKD management varied	
**Subtheme 3a:** patients understanding of their role in CKD management was limited to dietary and water intake, although some mentioned medication safety	**Quote 20:** (81, female, CKD 4) It was just one session, face-to-face and then a couple phone conversations. She just asked me a lot of questions and explained some stuff to me about what I should be eating and how I should be drinking more fluids and that, you know…I did [feel that the education sessions were helpful], because I’d watch what I was eatin’, like, you know, eatin’ low sodium stuff…not real high sodium lunch meat. And then I have been drinking more water…
	**Quote 21** (male, 78, CKD 3b) I think for the most part it was diet… what’s good for you, what’s not good for you, watch the salt… cook, buy food without having the sodium in it… about food to get and what to look for, when you do go to buy… I just remember mostly what she said about the food and, of course, very important that he drinks water…
	**Quote 22** (female, 73, CKD 3b) We’ve talked about the medication I’m on…my doctor advised to take me off of a certain medicine because it might be damaging my kidneys…
	**Quote 23** (male, 64, CKD 4) Told me I shouldn’t be taking ibuprofen. Switch to Tylenol.
**Subtheme3b:** knowledge of health implications was an important factor influencing interest in gaining more understanding of kidney disease and learning self-management skills	**Quote 24** (female, 73, CKD 3b) If I would get worse, I might have to go on dialysis. I might also need a kidney transplant. I’m hoping to take good care of myself so that I don’t advance to those stages.
	**Quote 25** (female, 84, CKD 4) I don’t know if there’s a way to reach people earlier… when they’re in stage 1 or stage 2. twenty-year-olds walking around in stage 1. Or maybe education for people in general. The way people know how what are the signs of a heart attack. Whatever it is that you need to do when you’re younger, could help people when they’re older.
	**Quote 26** (male, 67, CKD 3b) With the loss of the kidney, I have to watch my diet… the fear of the other kidney shutting down… The fear of maybe going on dialysis and stuff.
**Theme 4:** education sessions were well accepted and patients wanted more frequent and individualized sessions	
**Subtheme 4a:** patients appreciated education sessions which helped them increase their knowledge and alleviate anxiety	**Quote 27** (female, 84, CKD 4) Helped me to have an understanding of how to care for the kidneys… once you hear the words stage 4, there’s tremendous amount of fright and concern. Because you think it’s the same as stage 4 cancer or something like that… we started working about doing things like eliminating, any meds that…would influence the kidneys. Working with them on drinking more water…we found out that there were symptoms like shortness of breath to watch out …And so that eliminated a lot the panic and worry about hearing those words: stage 4.
	**Quote 28** (female, 73, CKD 3b) Following the suggestions that they gave me about… taking my blood pressure medicine like I’m supposed to, drinking lots of water, liquids every day, exercise…
	**Quote 29** (male, 68, CKD 4) The kidney sessions that I have gotten so far was helpful to let me know the best diet that I need to eat… exercise and all the other things that would help reduce, me from going to stage 5 or from becoming worse.
	**Quote 30** (female, 84, CKD 4) Knowledge is power… for example there are, I think, one of two medicines, completely were eliminated because… the benefits didn’t outweigh the costs to the kidneys… [The educator] worked with me on what to do diet-wise.
	**Quote 31** (male, 75, CKD 3a) Yes… I started thinking about kidneys rather than not thinking about them at all.
	**Quote 32** (male, 76, CKD 3b) It was informative. It was something that, you know, something that opens your eyes and think about it,
**Subtheme 4b:** patients suggested improvements in education sessions.	**Quote 33** (female, 72, CKD 3b) It wasn’t individualized enough… what I should be practicing or doing to try to prevent dialysis …
	**Quote 34** (male, 57, CKD 3a) Cuz a lot of people they see it in paper it’s a lot more…informative and helpful
	**Quote 35** (male, 64, CKD 4) I imagine doing a bit more explaining to people who don’t know… I’m a simple guy…So I don’t understanding legal mumbo jumbo.
	**Quote 36** (female, 80, CKD 3b) I was satisfied with the one that I had, although, perhaps, can you explain kidney disease in terms that people understand; exactly what does cause it?… And how it progresses, and what people should look out for?
	**Quote 37** (male, 68, CKD 3b) … list of things that you can do to improve, like, these are definite things that you have to do…like a shopping list of what you have to do, and to stay in line and make it a little more simplified for someone who’s not in the medical field.

*Note:* Quotes are referenced by numeric code throughout the text.

Abbreviations: CKD, chronic kidney disease; PCP, primary care provider.

### Theme 1. Patients Were Supportive of Collaborative Comanagement Support From Nephrologists to Their PCP

Patients wanted their PCPs to seek nephrologist input and maintain close communication. Patients trusted their PCP to identify worsening kidney function and seek consultation or refer them to a nephrologist if and when the need arose (quote 1) Patients also appreciated the value that every specialist contributes in their overall health care. The training and skill that a nephrologist could offer beyond a PCP’s toolkit were emphasized and patients preferred and believed that it would be beneficial if their PCP discussed management with the nephrologist. They wanted their PCP and specialists to talk with each other to comanage the coexisting diseases (quote 2). Although patients wanted nephrologist input, at the same time they expressed frustration with the burden of an in-office specialist visit and were uncertain of its utility beyond seeing their PCP (quote 5).

### Theme 2. Education Sessions had Variable Effect on Improving patients’ Understanding of CKD and its Health Implications

#### Spectrum of Knowledge About CKD and its Health Implications

Although all patients had received standard CKD education, patients had varied levels of understanding about the role of kidneys and what CKD meant. Some expressed a good understanding of CKD, but almost one-third were uncertain or reported a vague understanding of CKD but could not elaborate. Patients expressed a wide spectrum of understanding about the severity and etiology of their CKD. Many patients did not grasp how it affected their health and did not comprehend the future impacts. Many did not believe that it affected their health at all (quote 6). Some were aware that it can result in needing dialysis or kidney transplant, only a few mentioned it can cause death, and none mentioned the increased risk of cardiovascular disease (quote 7). Many patients associated lower urinary tract symptoms such as urgency and nocturia or back pain as symptoms because of CKD (quotes 8 and 9).

#### Poor Awareness of CKD and Low Priority

For some, CKD seemed to be low priority because they were not having symptoms, or because of other problems (quote 16). Some perceived CKD to be a low priority for PCPs and not usually discussed, but noted on after-visit documentation (quote 17).

### Theme 3. Patients’ Self-Efficacy and Understanding of CKD Management Varied

#### Patients Understanding of Their Role in CKD Management was Limited

Most patients recalled that dietary intake was important, but comments were not specific apart from advice to lower their salt intake, eat the right foods, and drink more water to flush their kidneys (quotes 20 and 21). Some patients discussed being told medications to avoid, such as nonsteroidal anti-inflammatory drugs (quotes 22 and 23).

#### Awareness of CKD Health Implications was an Important Factor Influencing Interest in Learning More About Kidney Disease and Self-Management Skills

Those who conveyed the long-term effect of CKD on their health, were interested to learn more about CKD, and self-management skills to maintain their kidney health. Those who knew that CKD could lead to dialysis and what dialysis entails expressed wanting to do what is necessary to avoid dialysis (quote 24). Patients supported increasing public general awareness of kidney disease, comparable to the public’s understanding of heart disease, and discussions about CKD at earlier stages to raise awareness of kidney disease and ways to prevent kidney function decline (quote 25).

### Theme 4. Education Sessions Were Well Accepted, and Patients Wanted More Frequent and individualized sessions

#### Patients Appreciated Education Sessions, Which Helped Them Increase Their Knowledge and Alleviate Anxiety

About 2 of 3 of the study participants gave a positive response when asked if learning about kidney disease helped them understand the CKD stages, and proactive steps could improve their kidneys and overall health (quotes 29 and 30). Most patients said that they would recommend the educational sessions to other patients with CKD.

#### Patients Suggested Improvements in Education Sessions

Patients wanted the sessions to occur more frequently and on a regular basis, and desired more explicit information on actionable next steps specifically individualized for them (quote 33). Patients also expressed wanting education to use simple language to explain kidney disease and what they can do (quotes 35 and 36). Patients suggested written hand-outs, such as a shopping list of things to do for kidney health (quote 37).

## Discussion

Overall, Kidney CHAMP PHM approach to CKD comanagement provided patients education and PCPs comanagement support that was accepted by the majority of patients. Evidence that the PHM approach was acceptable includes patient appreciation of additional CKD knowledge and comanagement support and oversight by a kidney specialist. However, despite additional education by a nurse, many patients still described a poor understanding of CKD causes and its relationship to comorbid conditions. The patient perspectives of Kidney CHAMP interventions highlight the need to reassess delivery of CKD education and tailor its content so that it is more effective at conveying CKD implications and treatment strategies.

Patients interviewed in OPTIMIZE CKD identified several positive attributes of Kidney CHAMP’s intervention. CKD comanagement by PCPs and nephrologists was valued by patients because of added confidence in nephrologists’ expertise on CKD. Kidney CHAMP provided a standard CKD education curriculum available on the National Kidney Foundation and Center for Disease Control websites to patients who may have never received focused CKD learning if only managed by their PCP. Interviewees also commented on the burden of travel to more office visits, which highlights the PHM benefit of telehealth educational visits and e-consults to facilitate PCP-nephrology comanagement, especially for patients without alarm signs of kidney disease (eg, hematuria, nephrotic syndrome, or rapidly progressing glomerulonephritis). Among all patient education components, counseling by a nurse on diet-related tips specific to fluid and sodium management seemed to stand out. This may be because patients perceive lifestyle counseling as more relatable, tangible, and actionable compared with CKD stages or discussions about prognosis. Future enhancements to CKD education could expand and individualize CKD diet and lifestyle change, which are powerful tools to combat CKD and related comorbid conditions.

We observed interesting differences in levels of understanding of their CKD despite all patient interviewees receiving standard CKD education by a trained nurse. Some patient respondents comprehended the interrelationship of kidney function with other comorbid conditions, whereas others attributed nonspecific urinary tract symptoms to kidney disease or the lack of symptoms to the absence of disease. Other patients described kidney disease is just something that happens, which reflects a misconception that CKD progression is unavoidable and unmodifiable. Whether these perceptions stemmed from differences in health literacy or from other factors, including personal beliefs, misinformation, or an oversimplification and low prioritization of CKD by their health care providers is uncertain. However, future studies may focus on examining how age, CKD stage, educational attainment, health literacy, and CKD education provider type influence patient comprehension of CKD education. Moreover, future work should also evaluate how education can be tailored to individual’s health literacy and needs.

Although most CKD education programs aim to fulfill patients’ desire to know more about kidney disease, they may fall short in the translation of knowledge into kidney health promoting action. Our patient interviewees provided valuable input on ways to improve CKD education. Interviewees expressed uncertainty about how to prevent CKD progression, highlighting that fact sharing does not clearly translate to concrete actionable steps to promote kidney health. Thus, we propose future considerations for CKD education based on interview feedback, such as the use of educational material such as a checklist of self-management tips and an interactive workshop series with lifestyle applications (Table 3). Content of educational material can be drawn from previous survey data on the needs and preferences in CKD education published by Allen et al.^20^ The leading educational topics were as follows: (1) definition of CKD, (2) definition of creatinine and GFR, (3) kidney diet, and (4) ways to slow the progression of kidney disease. However, in general, CKD education should use simple language and recommendations for lifestyle change should be individualized to each person.

**Table 3:** Lessons Learned From Patient Interviews and Future Directions to Improve Kidney CHAMP Interventions

Intervention and Goal	Lessons Learned	Future Directions
Nephrology e-consult Goal: Provide nephrology guidance to primary care providers (PCP) on individualized guideline-concordant CKD management using pharmacist support for medication reconciliation	Goal met Patients expressed general support for collaboration between PCP and nephrologist, and perceived value in the expertise a specialist could offer, and valued specialty kidney care without the travel burden of another office visit.	Continuing comanagement and nephrology specialist support for PCPs with ongoing follow-up
CKD education Goal: improve understanding of the following:	Goal met Most had some knowledge that dietary factors and certain medications affected kidney health.	1) To improve understanding of CKD and health implications
		• Distill education to fewer topics per session
		• Mode of education delivery should meet all learning styles
• CKD etiology and severity	Areas to improve	
• Health consequences of CKD	1) Poor understanding of:	2) To improve recall of education
• Recommended lifestyle and medication changes to help slow CKD progression	• CKD etiology and severity	• Develop educational series
	• Effect of CKD on health and individual risk for progression	• Apply constructive learning strategies
	2) Poor recall of other dietary and lifestyle recommendations	• Engage peer mentors or support person
	3) Limited implementation of lifestyle changes because of uncertainty of actionable steps	• Group meetings
		3) To identify actionable changes
		• Employ more individualized nutrition assessment and recommendations
		• Create specific, measurable, attainable, relevant, and time-specific goals

Abbreviations: CKD, chronic kidney disease.

Our qualitative study has several strengths, such as the inclusion of representatives of diverse populations, including those of low income, high comorbid condition severity, and advanced age. However, the OPTIMIZE CKD participants were sampled from Kidney CHAMP, which served Western Pennsylvania, which has an ∼80% non-Hispanic White population.^21^ Thus, there is a low representation of non-White individuals despite our best efforts to diversify the participant pool. Other limitations include recall bias, the inability to distinguish between patient CKD knowledge attained before and after the Kidney CHAMP intervention, and the lack of validated measures for health literacy, patient knowledge, and activation.

Overall, we learned that patients with CKD favored the PHM intervention, which connected them to a kidney specialist and educational resources. However, despite additional Kidney CHAMP resources, many participants were uncertain about the actionable steps to potentially delay progression of CKD or how to prepare for end-stage kidney disease. Future directions may include organizing CKD education into a series to reinforce key take-home points, diversify lessons, and enable participants to problem solve and share experiences with other people living with CKD.

## Supplementary Material

Supplementary File (PDF) Item S1
