# Supplementary material for: Patient Perceptions of a Population Health Management Program to Improve Kidney Care: Optimizing care in CKD
Source: Kidney Med. 2025 May 15;7(7):101025. doi: 10.1016/j.xkme.2025.101025 (PMC12213284; doi:10.1016/j.xkme.2025.101025)
Supplement: Supplementary File (PDF) — Item S1 [file mmc1.pdf]

Item S1. OPTIMIZE-CKD Patient Interview Guide

1. First, I'd like to ask some questions about how you understand chronic kidney disease. Could you describe CKD as you understand it?
   Probe as necessary:
   i. Could you describe for me what causes the disease?
   ii. Do you experience any symptoms that you think are from kidney disease?
   iii. How were you diagnosed?
   iv. How does CKD affect your health now?
   v. What is your understanding of how it could affect your health in the future?

2. My understanding is that you received some education sessions about kidney disease. Tell me about the kidney education sessions that you had. What were they like?
   i. Do you feel that the kidney education sessions were helpful for you? Why or why not?
   ii. When did you complete them?
   iii. Were they on the phone, on video call, or face-to-face?

3. Did learning about kidney disease help you understand how you can improve your kidney health and prevent dialysis? How so?

4. Were the education sessions interesting? Why or why not?

5. What questions did you have for the nurse? Was he/she able to answer all of your questions?

6. How could we improve the sessions?

7. Did you receive written information at or after the education sessions?
   i. Did you read the written information? If you didn't, why or why not?
   ii. Was it easy to understand?
   iii. Is there any way you would change or improve it?

8. Were you able to use/put into practice the recommendations that the nurse gave regarding kidney health?
   i. If not, what do you think kept you from using them?

9. Did the education sessions provide you with any tips to improve kidney health? If so, what were they?

10. Assuming that face-to-face sessions were safe, would you prefer these sessions as face-to-face visit, or phone or video-call?

11. How often would you like to have these sessions?

12. What was the scheduling process for the sessions like?

    i. Are there any changes you would make to the scheduling process?

13. Would you recommend the sessions to other kidney diseases patients?

14. Did you talk to a pharmacist about your medications? If so, was talking to the pharmacist helpful to manage your medications? Why or why not?

15. Has your PCP ever recommended seeing a nephrologist, or kidney doctor? Would you prefer to see in person or by tele-visit?

    i. Do you think it would be useful for your PCP to discuss your case with a kidney doctor? Why or why not?

16. Has your PCP ever recommended a treatment for your CKD that you decided not to take? If so, what was it, and what made you decide against it?

17. Those are all the questions I have for you today. Is there anything we didn't cover that you think we should know?
